# Exploring the link between self-management of migraine and emotional wellbeing: a cross-sectional study of community-dwelling migraine sufferers

**DOI:** 10.1186/s12883-024-03535-0

**Published:** 2024-01-27

**Authors:** Nicola Filzmoser, Iman Webber, Gabriele Kerr, Aos Alaa, Marie Iine El Asmar, Manisha Karki, Eva Riboli-Sasco, Austen El-Osta

**Affiliations:** Department of Primary Care & Public Health, Self-Care Academic Research Unit (SCARU) Imperial College London, Charing Cross Hospital, London, W6 8RF UK

**Keywords:** Migraine, Self-care, Lifestyle change, Lifestyle medicine, Self-management, Headache, Self-care, Prevention, Emotional wellbeing

## Abstract

**Background:**

Globally, an estimated 14% of adults live with migraine disease which impacts their physical, emotional and social wellbeing. To target the disease comprehensively, research recommends a multidisciplinary approach to migraine management. Yet, at present, migraine management primarily centers around pharmaceutical treatments. The aim of this study was to investigate the extent to which emotional awareness could influence the uptake of self-care behaviours of community-dwelling adults with migraine.

**Methods:**

A cross-sectional online survey explored personal experiences with migraine disease and strategies or behaviours to manage migraine attacks. Chi-squared tests were used to investigate differences in ratings of migraine prevention and management strategies between users and non-users of the strategies. Univariable logistic regressions were used to assess the effectiveness of self-care behaviours to manage or prevent migraine attacks.

**Results:**

We surveyed 170 community-dwelling adults with migraine in the United Kingdom, Austria, Germany and the United States. Most (85%) respondents had experienced migraine for over five years, where 42% of attacks usually lasted several days. Whereas we did not differentiate between diagnosis by a neurologist or self-diagnosis, the most common diagnoses in the cohort were migraine without aura (38.9%) and migraine with aura (29%). Staying hydrated was the most popular preventative strategy (87%), 70.2% used prescription medication and 64.9% changed their diet and/or supplements. Almost all ( 92.4%) respondents stated that their mood or emotions could trigger their migraine attacks. Keeping a headache or mood diary was the lowest-rated prevention strategy and was rated as "probably ineffective" or causing "no change" in preventing migraine attacks. Over a third (39.7%) kept track of their physical wellbeing and symptoms. Reasons stated for tracking symptoms included to identify triggers (65.8%), show reports to a healthcare professional (59.6%), understand when they must take medication (48.1%), track improvements (67.3%) or deteriorations (67.3%).

**Conclusions:**

Migraine management is dominated by pharmaceutical management for acute pain attacks and lifestyle changes for managing migraine long-term. Perception of the effectiveness of those techniques is high, whereas perception of interventions that target the emotional or psychological components of chronic pain management (keeping a mood diary, and mental health support) is mixed. There exists a gap between the recommended biopsychosocial approach and the current state of migraine management.

**Graphical Abstract:**

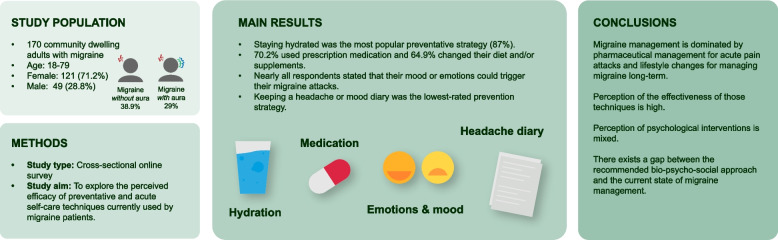

**Supplementary Information:**

The online version contains supplementary material available at 10.1186/s12883-024-03535-0.

## Background

Chronic pain, defined as pain that persists or recurs for longer than three months [1], affects a quarter of the UK population [2]. The Global Burden of Disease Study 2016 recognised the high prevalence of pain and pain-related disease as a major contributor to disability and disease burden worldwide [3]. Chronic pain has been shown to have a significant impact on people’s overall quality of life [4].This significant impact can also be found in headache disorders, which are among the most common diseases worldwide. Migraine, a primary headache disorder, is one of the leading causes of years lived with disability among adults [5]. The estimated global prevalence of migraine is 14% in adults [6]. Aside from debilitating physical symptoms, migraine has a significant impact on a person's daily functioning, quality of life, social and emotional wellbeing, employment and family life [7–10].

Over the past century, our understanding of and perspective on chronic pain has shifted from a biomedical perspective which regards pain as an objective physical event, to a biopsychosocial perspective which considers the interplay of physiological, psychological, social, cognitive and behavioural factors present in an individual's pain experience [11]. While certain contributing factors in chronic pain are deemed non-modifiable (i.e., age, sex, history of trauma or injury, or heritable/genetic factors) other modifiable contributing factors play an important role including pain, mental health, physical activity, sleep and nutrition [12]. In addition to genetic predisposition, behavioural and psychological traits may adversely affect the progression of the disease and how pain is processed [13]. Self-management behaviours and lifestyle modification targeting sleep, diet, stress or the work environment could offer a significant contribution to the experience and trajectory of chronic pain [12, 13]. Migraine is often influenced by comorbidities such as back pain, anxiety and depression or diabetes, as well as individual psychological characteristics. A management approach addressing migraine and simultaneously comorbidities is considered to positively impact the outcome on the individual’s health status. However, also in individuals free of psychiatric comorbidities, specific behavioural and psychological factors might occur which need to be addressed for improved management [14, 15].

Internationally, guidelines for migraine treatment support this multidisciplinary approach, which includes a combination of acute and preventative pharmacological treatment, lifestyle modification and the use of evidence-based behavioural interventions [16, 17]. The routine use of these self-care techniques and behavioural interventions in headache management is low due to factors such as missing knowledge among healthcare professionals [18]. Additionally, headache patients consider behavioural interventions only suitable for those who have previously not benefited from pharmacological treatment options or have high-severity symptoms [18]. Across migraine management, the sustained adoption and adherence to lifestyle or behavioural modifications, including nutrition or use of medication, pose challenges [19, 20]. To address this, several dimensions of self-care which act on the health system level and individual need to be considered, which can be broadly grouped into [1] self-care activities (self-awareness, health literacy) and [2] self-care behaviours (delivery, activation, and behaviour change) [21]. In the case of migraine, as with the management of other chronic conditions, self-care activities and behaviours are primarily centred around physical symptoms of pain, whereas behaviours including a doctor's visit or taking a pill are considered to be responses to physical pain [22].

From the biopsychosocial perspective of chronic pain, this one-sided approach lacks the integration of emotional and social factors and the influence this would have on the choice of self-care activities and behaviours which should be present to address acute and non-pain situations. Using personal mood and emotion-tracking smartphone applications can increase an individual's emotional awareness [23]. In the case of migraine sufferers, using mood diaries and emotion tracking could help them become more aware of their emotions and would be more likely to react to them using self-management approaches. Available support for self-management of migraine, including the use of headache- or migraine-tracking apps, follow this approach but traditionally favour the tracking of physical symptoms of migraine over the impact of emotions in their assessment [24, 25]. Further, while these apps may enhance the recording of and the user's reflection on emotions, they do not routinely provide guidance for behavioural responses to emotions and social situations [26].

### Study objectives

The purpose of this study was to explore the perceived efficacy of preventative and acute self-care techniques currently used by migraine patients. We also sought to examine the extent to which emotional factors are integrated into the choice or management of migraine.

## Methods

### Study design

We conducted a descriptive cross-sectional online survey of migraine sufferers using purposive sampling. The electronic survey published on the Imperial College London Qualtrics platform was open for 8 months (July 2021 to February 2022) and could be accessed by anyone with the link. Information about the study was shared with migraine charities as well as through the personal networks of the co-investigators. Initial contact was not made with respondents prior to this email. Study information inviting individuals to contribute to a study that investigated the extent to which emotional awareness influences self-care behaviours of adults with migraine was disseminated, including the Participant Information Sheet (PIS) and link to the survey. Inclusion criteria were only validated in the survey asking participants if they had migraine disease. We did not specify diagnosis by a neurologist or self-diagnosis nor a specific type of migraine as exclusion criteria. The researchers' personal and professional networks were also mobilised to respond and further disseminate the eSurvey among eligible participants. The PIS included information regarding the study's aims, the protection of participants' personal data, their right to withdraw from the study at any time, which data were stored, where and for how long, who the investigator was, and the purpose of the study and survey length. Participants were informed that this was a voluntary survey without any monetary incentives. We highlighted that their participation offered collective benefits by advancing knowledge in the area. We offered participants access to the study findings at a later stage.

### Electronic survey

The Qualtrics survey (Version XM) contained a total of 26 questions displayed on one page. The Qualtrics website has first-party cookies and allows third parties to place cookies on devices and automatically capture responses. The survey was accessible using a personal computer or smartphone to anyone with a link (open survey). Questions regarding the demographic characteristics of the users included information on gender, age, ethnicity, educational level, marital status, country of residence and employment status. Participants could review their answers before submitting them. All data collected through the survey were anonymised and not personally identifiable. The online survey’s technical functionality and usability were tested and piloted with a small group of individuals before being published. Participation in the survey was voluntary, and consent to participate in the study was sought in the first question of the survey.

The survey contained conditional questions that appeared if the respondent stated they track their emotional and/or physical wellbeing and symptoms so that they could input further information about their personal experiences. Single-choice questions evaluated the participants' experience with migraine disease (e.g., frequency or type). Personal self-care techniques for migraine attack prevention and acute management were evaluated in multiple-choice questions with common self-care techniques as suggested answer options. Participants were asked to state the extent to which they thought the listed self-care techniques were effective. Additional questions explored the participants' perception of how emotional wellbeing was related to their migraine disease and its management and whether they tracked their physical and emotional wellbeing. The survey is included in Supplementary File 1.

### Data handling

The data collected were stored on the password-protected Imperial College London secure database, and only the team researchers could access the eSurvey results. Data were cleaned to exclude respondents who did not provide tick-box consent, or who exited without submitting their response. Non-submitted responses were excluded as these lacked answers to key questions on participant demographics and experience, prevention, or management of migraines. IP addresses of survey responses indicated no duplicated participants.

### Statistical analysis

Respondent characteristics were described using frequencies and percentages for categorical variables and median with interquartile range (IQR) for continuous variables. Chi-squared tests were conducted to explore potential associations between ratings of each prevention and management strategy and being a user of the strategy. The Holm correction for multiple tests was applied to the resulting *p*-values of statistical tests. An adjusted *p*-value < 0.05 denoted statistical significance. All analyses were performed using R version 4.1.2. The quality of the survey was assessed by completing the Checklist for Reporting Results of Internet E-Surveys (CHERRIES) [27].

### Ethics

The study received a favourable opinion from Imperial College Research Ethics Committee (ICREC # 21IC6990). Participants consented to take part in the survey.

### Patient and public involvement

No patient was involved.

## Results

### Demographic profile of respondents

The electronic survey received 170 total responses. Only 131 (77.1%) of the total responses were complete, emanating from the United Kingdom (84.0%), Germany (2.3%), Austria (1.5%) and the United States (12.2%); Table 1. The majority (92.4%) of respondents were female. Respondents had a median age of 39 years (IQR 29 – 51) with a range of 18 to 74 years. Eighty-three percent were educated to a university degree or higher, and 60.3% were employed. More than half (51.9%) were married, 20.6% were in a domestic relationship, and 17.6% were single. The majority (90.8%) identified as white ethnic background.

**Table 1:** Respondent characteristics

	***N***	**(%) including missing**	**(%) excluding missing**
Age (years), median (IQR)	39.0 (29.0 – 51.0)		
**Gender**			
Female	121	92.4	92.4
Male	8	6.1	6.1
Other	2	1.5	1.5
**Country**			
United Kingdom	110	84.0	84.0
Germany	3	2.3	2.3
Austria	2	1.5	1.5
United States	16	12.2	12.2
**Ethnicity**			
White	119	90.8	95.2
Mixed/Multiple ethnic groups	2	1.5	1.6
Asian/Asian-British	1	0.8	0.8
British Black/African/Caribbean	1	0.8	0.8
Other	2	1.5	1.6
Missing	6	4.6	-
**Education**			
A levels/ College	13	9.9	10.0
Secondary School	8	6.1	6.2
University Degree or higher	109	83.2	83.8
Missing	1	0.8	-
**Employment**			
Employed	79	60.3	61.2
Self-employed	17	13.0	13.2
Unable to work	20	15.3	15.5
Unemployed	7	5.3	5.4
Retired	6	4.6	4.7
Missing	2	1.5	
**Marital Status**			
Divorced	7	5.3	5.4
Married	68	51.9	52.3
Separated	1	0.8	0.8
Widowed	3	2.3	2.3
In a domestic relationship	27	20.6	20.8
Single	23	17.6	17.7
Other	1	0.8	0.8
Missing	1	0.8	-
**Diagnosed migraine type**			
Migraine with aura	38	29.0	29.0
Migraine without aura	51	38.9	38.9
Hemiplegic migraine	11	8.4	8.4
Vestibular migraine	4	3.1	3.1
Abdominal migraine	1	0.8	0.8
Other type/ don’t know the type	17	13.0	13.0
No migraine diagnosis	9	6.9	6.9
**Migraine duration**			
30 min to an hour	2	1.5	1.5
Over an hour	26	19.8	20.0
The whole day	47	35.9	36.2
Several days	55	42.0	42.3
Missing	1	0.8	-
**Migraine frequency**			
Once or twice per year	5	3.8	3.8
Once or twice per month	34	26.0	26.0
Once or twice per week	34	26.0	26.0
Chronic (15 + days per month)	58	44.3	44.3

### Migraine characteristics and impact

Eighty-nine percent of respondents had experienced migraine for over five years. Whereas 26% of respondents experienced migraine once or twice per week, 26% once or twice per month, and 3.8% once or twice per year, 44.3% indicated they had chronic migraine (15 + days per month). At the time of survey completion, 77.1% of respondents had experienced their last migraine attack during the last seven days. Migraine attacks usually lasted several days (42.0%), the whole day (35.9%), or over an hour (19.8%). The most common diagnoses in the cohort were migraine with no aura (38.9%) and migraine with aura (29.0%). A quarter (25.2%) indicated they had been diagnosed with a different migraine type or that they did not know the type of migraine they experienced. Respondents were asked to rate on a scale of 0 to 5, where 0 is no effect, and 5 is an extreme effect, the extent to which migraine affected their physical, emotional and social wellbeing. An extremely detrimental effect on physical wellbeing with a score of 4 or more was most frequently reported (73.3%), compared to 64.9% for social wellbeing and 59.5% for emotional wellbeing.

### Migraine prevention

**Fig. 1:**
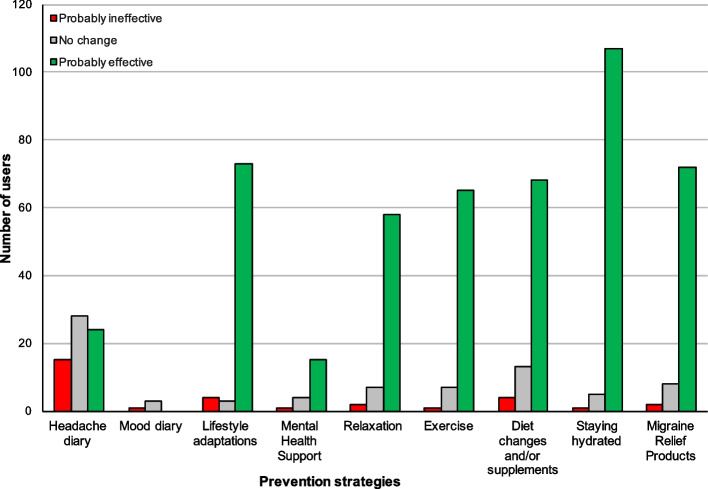
Rating of prevention strategies from users. Figure 1 shows the subjective rating of prevention strategies from those who actively use the strategy (= users)

**Fig. 2:**
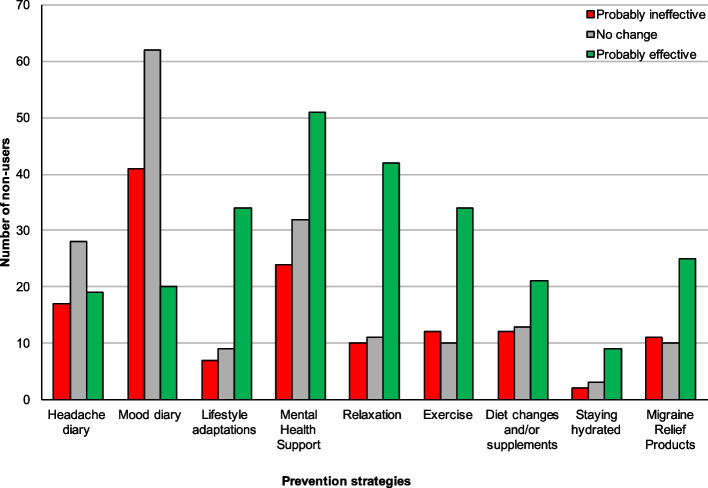
Rating of prevention strategies from non-users. Figure 2 shows the subjective rating of prevention strategies from those who have never used or have stopped using the strategy (= non-users)

Respondents were divided into "users" (Fig. 1) and "non-users" (Fig. 2) of each migraine prevention activity based on whether or not they had indicated they used the activity for the prevention of migraine attacks and maintenance of physical and emotional wellbeing. Users of each prevention strategy generally rated them more positively than non-users, except for "Headache Diary" which was viewed as ineffective by both users and non-users. Table 2 shows the subjective ratings of all prevention strategies in more detail.

**Table 2:** Rating of prevention strategies stratified by users and non-users of the strategy

Strategy	Rating	TOTAL, *n* (%)	Non-users	Users	*P* value
			***n* (%)**	***n* (%)**	
**Headache Diary**	Probably ineffective	32 (24.4)	17 (26.6)	15 (22.4)	1
	No Change	56 (42.7)	28 (43.8)	28 (41.8)	
	Probably effective	43 (32.8)	19 (29.7)	24 (35.8)	
**Mood Diary**	Probably ineffective	42 (33.1)	41 (32.3)	1 (25.0)	1*
	No Change	65 (51.2)	62 (48.8)	3 (75.0)	
	Probably effective	20 (15.7)	20 (15.8)	0 (0.0)	
**Lifestyle adaptations**	Probably ineffective	11 (8.5)	7 (13.7)	4 (5.0)	0.021
	No Change	12 (9.2)	9 (17.7)	3 (3.8)	
	Probably effective	107 (82.3)	34 (66.7)	73 (91.3)	
**Mental Health Support**	Probably ineffective	25 (19.7)	24 (21.6)	1 (5.0)	0.270
	No Change	36 (28.3)	32 (28.8)	4 (20.0)	
	Probably effective	66 (52.0)	51 (45.9)	15 (75.0)	
**Relaxation**	Probably ineffective	12 (9.2)	10 (15.9)	2 (2.9)	0.089
	No Change	18 (13.8)	11 (17.5)	7 (10.3)	
	Probably effective	100 (76.9)	42 (66.7)	58 (85.3)	
**Exercise**	Probably ineffective	13 (10.1)	12 (20.7)	1 (1.4)	< 0.001
	No Change	17 (13.2)	10 (17.2)	7 (9.6)	
	Probably effective	99 (76.7)	34 (58.6)	65 (89.0)	
**Diet changes and/or supplements**	Probably ineffective	16 (12.2)	12 (26.1)	4 (4.7)	< 0.001
	No Change	26 (19.8)	13 (28.3)	13 (15.3)	
	Probably effective	89 (67.9)	21 (45.7)	68 (80.0)	
**Staying Hydrated**	Probably ineffective	3 (2.4)	2 (11.8)	1 (0.9)	< 0.001
	No Change	8 (91.3)	3 (17.7)	5 (4.4)	
	Probably effective	116 (91.3)	9 (52.9)	107 (93.9)	
**Migraine Relief Products**	Probably ineffective	13 (10.2)	11 (22.9)	2 (2.4)	< 0.001
	No Change	18 (14.1)	10 (20.8)	8 (9.6)	
	Probably effective	97 (75.8)	25 (52.1)	72 (86.8)	

Percentages are shown as percent of respondents who were users or non-users of the specific strategy. Percentages may not add up to 100% as 'Unknown' categories are not shown. *P*-values correspond to those of Pearson's chi-squared tests. * There were too few users of the Mood Diary strategy to perform a chi-squared test; a Fisher’s exact test was used

The most popular migraine prevention strategy was 'staying hydrated', with 87% of respondents selecting that they partake in the strategy (Fig. 1). The next most popular prevention strategies, in descending order, were taking prescription medication (70.2% of 131 respondents selected), diet changes and/or supplements (64.9%), using migraine relief products (63.4%), and lifestyle adaptations such as regular breaks or avoiding screen time (80%).

Staying hydrated was the highest-rated strategy by its users with nearly 94% describing it as "probably effective". The next best-rated prevention strategies by user rating were lifestyle adaptations and exercise, with 91.3% and 89% of their respective users rating them as "probably effective". Keeping a mood diary (3.1%) was the least frequently selected strategy for the prevention of migraine attacks. Keeping a headache or mood diary was the lowest rated ("probably ineffective") prevention strategy amongst both users and non-users of the strategies (Figs. 1 and 2).

Although mental health support "Counselling, Cognitive behavioural therapy, other mental health support" was the second least frequently selected strategy for migraine prevention (15.3%), it was highly rated by its users, with 75% of respondents who used this strategy (*n* = 20) describing it as "probably effective" at preventing migraine.

### Acute migraine management strategies

**Fig. 3:**
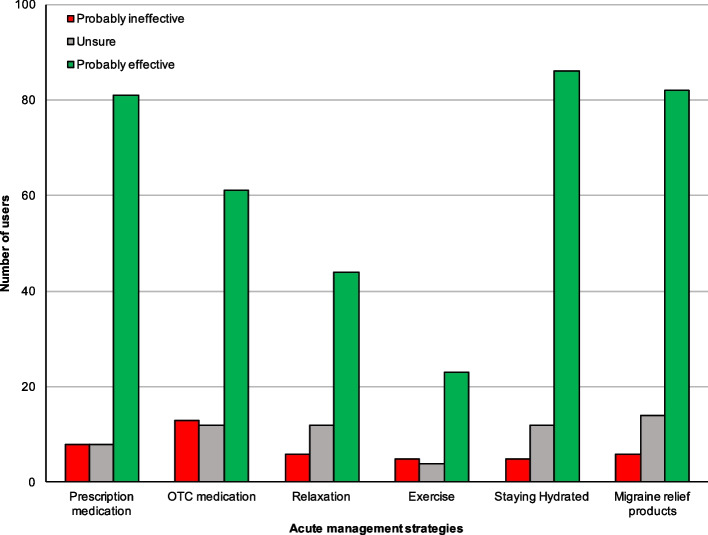
Rating acute management strategies from users. Figure 3 shows the subjective rating of acute management strategies from those who actively use the strategy (= users)

**Fig. 4:**
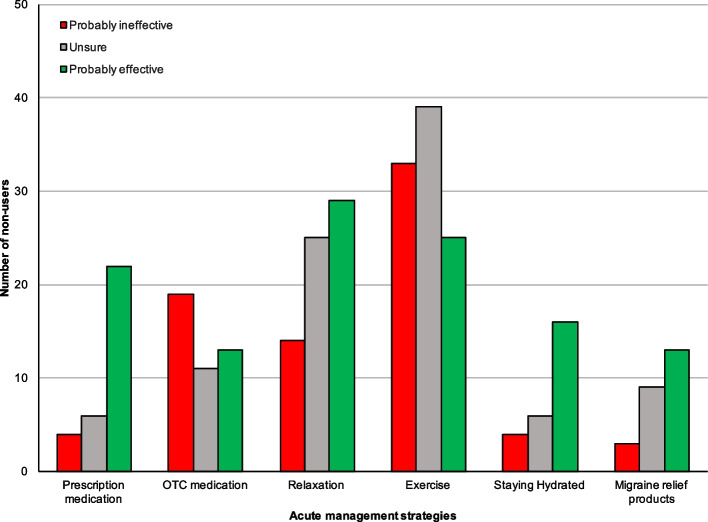
Rating acute management strategies from non-users. Figure 4 shows the subjective rating of acute management strategies from those who have never used or have stopped using the strategy (= non-users)

Respondents were divided into "users" (Fig. 3) and "non-users" (Fig. 4) for each migraine management activity based on whether they used the activity to manage signs and symptoms of migraine when they occur. Most management strategies were rated positively by users, whereas non-users were more often doubtful as to the effectiveness of each strategy. Table 3 shows the subjective ratings of all prevention strategies in more detail.

**Table 3:** Rating of acute management strategies stratified by users and non-users of the strategy

Strategy	Rating	Total, n (%)	Non-users, n (%)	Users, n (%)	*P* value
**Prescription medication**	Probably effective	103 (79.8)	22 (66.7)	81 (82.7)	0.241
	Probably ineffective	12 (9.3)	4 (12.12)	8 (8.2)	
	Unsure	14 (10.9)	6 (18.18)	8 (8.2)	
**OTC medication**	Probably effective	74 (57.4)	13 (28.9)	61 (70.9)	< 0.001
	Probably ineffective	32 (24.8)	19 (42.2)	13 (15.1)	
	Unsure	23 (17.8)	11 (24.4)	12 (14.0)	
**Relaxation**	Probably effective	73 (56.2)	29 (42.0)	44 (71.0)	0.037
	Probably ineffective	20 (15.4)	14 (20.3)	6 (9.7)	
	Unsure	37 (28.5)	25 (36.2)	12 (19.4)	
**Exercise**	Probably effective	48 (37.2)	25 (25.3)	23 (71.9)	< 0.001
	Probably ineffective	38 (29.5)	33 (33.3)	5 (15.6)	
	Unsure	43 (33.3)	39 (39.4)	4 (12.5)	
**Staying Hydrated**	Probably effective	102 (79.1)	16 (59.3)	86 (82.7)	0.117
	Probably ineffective	9 (7.0)	4 (14.8)	5 (4.8)	
	Unsure	18 (14.0)	6 (22.2)	12 (11.5)	
**Migraine relief products**	Probably effective	95 (74.8)	13 (48.2)	82 (78.9)	0.042
	Probably ineffective	9 (7.1)	3 (11.1)	6 (5.8)	
	Unsure	23 (18.1)	9 (33.3)	14 (13.5)	

Percentages are shown as percent of respondents who were users or non-users of the specific strategy. Percentages may not add up to 100% as 'Unknown' categories are not shown. Abbreviations: *OTC* over the counter, *P*-values correspond to those of Pearson's chi-squared tests

The most popular acute management strategies by several users were 'staying hydrated' and 'use of migraine relief products', with both being used by 104 (79.4% of 131) users. Taking prescription medication was also popular, with 74.8% of respondents selecting it as a management strategy.

Staying hydrated and taking prescription medication were the most highly rated management strategies, with 82.7% and 82.7% of their respective users rating them as "probably effective" at managing signs and symptoms of migraine in that same order. Using a mood diary to manage signs and symptoms of migraine attacks was the least popular strategy, with only 4 (3.1%) respondents selecting it. Exercise and OTC medication received the highest proportion of "probably ineffective" ratings of all management strategies as rated by non-users, users of the strategies much more positively, with 71.9 and 70.9% rating them as "probably effective", respectively.

### Self-tracking of wellbeing and symptoms

Twenty-two respondents (16.8%) tracked both their physical and emotional wellbeing, whereas 73 (55.7%) stated they neither tracked their physical nor emotional wellbeing and symptoms.

Over a third (39.7%) of respondents stated they kept track of their physical wellbeing and symptoms. Respondents used the data they collected about their physical wellbeing and symptoms to identify triggers (65.8%), show reports to a healthcare professional (59.6%), understand when they must take medication (48.1%), track improvements (67.3%), or to track deteriorations (67.3%).

Nearly all (92.4%) respondents stated that their mood or emotions could be a trigger for their migraine attacks.

Mood or emotions were a trigger most or all of the time for 36.6% of respondents. Less than a quarter (19.1%) of 131 respondents stated they keep track of their emotional wellbeing and symptoms. Respondents that tracked emotional symptoms often tracked physical symptoms – 88% of respondents who tracked their emotional wellbeing and symptoms also tracked their physical wellbeing and symptoms, but only 42.3% of respondents who tracked their physical wellbeing and symptoms also tracked their emotional wellbeing and symptoms.

Respondents used the data they collected about their emotional wellbeing and symptoms to identify triggers (76%), show reports to a healthcare professional (44%), understand when they have to improve their emotional wellbeing (72%), track improvements (52%), and track deteriorations (13%). Migraine duration, frequency, and type were not found to be associated with the likelihood of tracking emotional wellbeing and symptoms.

## Discussion

To our knowledge, this is the first study that sought to understand the perceived efficacy of preventative and acute self-care techniques currently used by migraine patients, and to examine the extent that emotional factors are integrated into choice or management of migraine. The overall aim of the paper was to investigate the extent that emotional awareness influences self-care behaviours of community-dwelling adults with migraine.

## Key findings

### Focus on physical symptoms

Findings revealed that participants rated the impact of migraine to be similar on their physical, emotional, and social wellbeing, but this was not reflected in their choice of prevention and acute migraine management strategies, which are mainly focused on treating physical symptoms. The most popular choices of those strategies were staying hydrated or taking prescription medication. This is consistent with the findings of Probyn et al., (2017), which showed that non-pharmacological self-management interventions for migraine are more effective than usual care and in reducing pain intensity, mood and headache-related disability.

A hypothesis to explain the more positive rating of strategies by users compared to non-users is the difficulty of understanding a strategy’s value if you have never used it yourself. Additionally, non-users could consider these strategies as an additional burden to their management.

Behavioural and cognitive behavioural therapy (CBT) have been shown to be effective in reducing pain, distress, pain behaviour in chronic pain patients and in improving daily functioning [1, 2]. Although in our study only 20 participants reported using mental health support including CBT as a strategy for migraine prevention, it was highly rated as 'probably effective' by users of this self-management technique. Matsuzawa et al. [3] previously explored the barriers to behavioural treatment (e.g., biofeedback, CBT, relaxation techniques) adherence which could be a barrier for the existing target group as well. Further because migraine is known to arise from an interaction between biological, psychological and lifestyle factors, migraine disease puts a physiological and psychological strain on those affected. Psychological factors in particular might aggravate the disease progression, exposing individuals affected to a negative cycle [13].

### Barriers to interventions

Despite the promising efficacy of behavioural interventions which had fewer side effects than pharmacological treatment, a key barrier to the uptake and adherence to behavioural interventions was related to attitudes and beliefs towards the interventions [3]. Another barrier highlighted was the lack of knowledge or unawareness of headache triggers which could be grouped into a number of categories as follows: [1] 'avoidable' triggers (e.g., alcohol); [2] 'unavoidable and unmanageable' triggers (e.g., weather); and [3] 'unavoidable but manageable' triggers (e.g. stress). Monitoring and managing triggers could help patients increase their headache control which in turn can lead to improved treatment adherence [3].

Rosignoli et al. (2022) [13] highlight that a biopsychosocial approach that considers psychological mechanisms, social and lifestyle factors in addition to biological factors, is already widely used in chronic pain management. However, in migraine management the biomedical approach is dominant. A potential barrier to adopting the approach is the incomplete knowledge of migraine pathophysiology. Additionally, the variability of behavioural interventions for each individual is higher than in pharmacological solutions, leading to difficulty in measuring their effectiveness. The high prevalence of migraine would demand a high level of resources which in the current healthcare system cannot be provided to all individuals with migraine who would require them.

A recent study from the German Migraine and Headache Society (DMKG) highlighted that headaches show higher impact in individuals with a low socioeconomic status which needs to be further considered in the selection and availability of new management solutions [28].

### Use of a diary to monitor/help prevent migraine

Pertinently, a diary to monitor mood and physical symptoms (e.g., Headache or symptoms) was only used by 67/170 (39.4%) of respondents and this self-care strategy was rated as 'probably effective' by only 35.8% of diary users. Conversely, the use of mood diaries for the prevention of migraine attacks was the least used strategy with only four respondents rating the effectiveness as either 'probably ineffective' or 'no change'. A clear focus is put on tracking physical symptoms, improving one's understanding of emotional or mood factors influencing pain is uncommon and not considered to be an effective management strategy. The recommended biopsychosocial approach to chronic pain, including migraine, management, aims to address physiological as equally psychological, social, cognitive and behavioural factors in a patient's pain experience [4]. Physiological elements are usually managed primarily and tracked in headache diaries by a large number of migraine patients. Mood diaries could further support the biopsychosocial treatment approach by helping patients to learn about mood patterns, hence, being actively able to act to improve their mood [5]. Based on our study findings, we identified that only some diary users consider diaries to be ‘probably effective’. However, we did not define what ‘probably effective’ means in detail for a migraine diary. We can assume that participants rated the strategy in how it was effectively contributing to their migraine management. We cannot draw a conclusion as to whether users find diaries effective in learning about mood patterns. An in-depth analysis is needed to confirm this. An implication for clinical practice would be to use combined diaries that include mood and symptom tracking and show their relationship in summarised reports. This might help patients but also clinicians to understand challenge areas which could be e.g. emotional stressors.

### Limitations

The principal limitation of this survey was that 92.4% of survey respondents were women. A reason for this unbalanced gender distribution might be the prevalence of migraine, which current literature shows to be three to four times higher in women than in men. A larger sample in future studies will be important to understand if this effect is due to the sample size instead.

Overall, the number of participants does not allow for conclusive statements on whether the lack of use of mood diaries and the belief that they are ineffective correlates with a low number of users of mental health services. While many factors such as availability or cost will impact the choice of interventions, a potential area for further investigation is how increased emotional awareness (through e.g., the use of mood diaries) would influence the use of mental health support in migraine prevention and acute management.

Another key limitation of this study is related to the small sample size, but it is generally difficult to recruit for such community-facing studies unless migraine patients are approached via the healthcare provider.

Further, a convenience sampling strategy was used because we were unsure how many migraine sufferers would respond to the survey. Given that most studies fail because they are unable to recruit the desired sample, convenience sampling was chosen due to its accessibility and time efficiency and this approach is particularly useful for reaching specific groups online, such as migraine communities, and is well-suited for exploratory studies or when resources are limited. As such, we did not seek to perform a power calculation but instead recruited a pragmatic sample primarily due to the exploratory nature of the research, constraints in resources and time, and the need for real-world applicability. This approach allowed for broader inclusivity and greater feasibility, especially in preliminary investigations or when studying novel topics where effect sizes are uncertain. While this decision prioritizes practicality and real-world relevance, we acknowledge that this does not consider potential trade-offs in statistical precision and generalizability. Despite this limitation, we felt that using a pragmatic sample was justifiable given the aims and nature of our study.

To enhance the usability of the survey, self-care interventions were grouped within answer options. Follow-up explorations of individual interventions would be necessary. Also, users of prevention/management strategies are likely users because they have found that those strategies work for them. Non-users may be composed of both those who have never tried the strategy and those who have tried the strategy in the past and found it did not work for them and, hence, do not currently use the strategy.

Additional focus needs to be placed on the migraine frequency and how it relates to the acceptance of (new) self-care techniques. The burden of migraine disease can differ significantly depending on the frequency of migraine attacks. 3.8% of our respondents experienced migraine attacks once or twice per year whereas 44.3% indicated they had chronic migraine (15 + days per month, 8 of those with migraine symptoms). The impact on lifestyle modifications necessary such as social interactions or regular sleep schedules most likely vary a lot. In our analysis, we did not differentiate based on frequency in measuring the acceptance of self-care techniques. We can assume that those with a higher frequency already have a larger toolkit of migraine management strategies (acute, preventative, or self-care) which potentially could impact the willingness to add new strategies to their management. On the other hand, those with a low frequency might not be willing to engage in new strategies as the impact of the disease might be lower.

The topic of using a mood journal was only explored in relation to migraine. Asking for general use of (mood) journals could have given additional insights into patients' behaviour. Due to the low number of respondents who are using emotional diaries, it is difficult to conclude any feedback on their importance or benefit.

Pertinently, such phenotyping of patients can help in designing efficient clinical trials. For example, a patient who is already keeping a diary may not need to be randomized into a self-management intervention which utilizes such methods, and this may help streamline recruitment into the trial.

### Implications

The survey analysis suggested that migraine management is currently focused on pharmaceutical interventions and lifestyle changes such as staying hydrated. The aspect of emotional wellbeing is less integrated into patients' current coping and management strategies. However, mental health interventions are subjectively perceived as helpful by respondents, which allows the assumption that awareness is given. Future research would need to explore how this gap between awareness and actual implementation of e.g., mental health support in migraine management arises and how it can be overcome.

## Conclusions

Migraine management is dominated by an over-reliance on pharmaceutical interventions to manage acute pain attacks, whereas lifestyle changes are usually considered as a second option for managing migraine in the long term. Perceptions on the effectiveness of those techniques are high whereas perception of interventions that target the emotional or psychological component of chronic pain management (e.g., keeping a mood diary, and mental health support) are mixed. There exists a gap between what the latest research on chronic pain and migraine management suggests, a bio-psychosocial approach, and the current state of migraine management. Future research is necessary to identify causes and solutions for this gap.

### Supplementary Information

**Additional file 1.** Export of study survey.

## Data Availability

The datasets used and/or analysed during the study are available from the corresponding author on reasonable request.

## Abbreviations

CBT: Cognitive Behavioural Therapy
IQR: Interquartile range
OTC: Over the counter
