# Supplementary material for: Exploring the link between self-management of migraine and emotional wellbeing: a cross-sectional study of community-dwelling migraine sufferers
Source: BMC Neurol. 2024 Jan 27;24:47. doi: 10.1186/s12883-024-03535-0 (PMC10821553; doi:10.1186/s12883-024-03535-0)
Supplement: Supplementary file 1 — Additional file 1. Export of study survey. [file 12883_2024_3535_MOESM1_ESM.pdf]

Export of study survey

(SAME) Migraine & emotional wellbeing

Start of Block: Start page

Q1 Imperial College London Department of Primary Care & Public Health is conducting a qualitative study to assess self-care behaviours of young adults with migraine. We're keen to hear your views to learn about your experience and perspective. All information will be treated confidentially. Thank you for participating in this short (10 min) survey.

For more information please see the Participant Information Sheet.

Q2 Do you consent in taking part in this study? If yes, you understand that:

-You've read the Participant information sheet (above) and have had the opportunity to ask any questions you might have on the study.
-Your participation is voluntary & you are free to withdraw at any time.
-Imperial College London will have access to your records relevant to this research.
-Information collected may be used to support other research in the future.

○ Yes (1)
○ No (2)

Skip To: End of Survey If Do you consent in taking part in this study? If yes, you understand that: -You've read the Partic... = No

---

Q3 Do you suffer from migraine attacks / have migraine disease?

○ Yes (2)
○ No (1)

Q4 If yes, how long have you been experiencing migraines?

○ Less than a year (3)
○ 1-3 years (2)
○ 3-5 years (1)
○ >5 years (4)

© Imperial College of Science, Technology and Medicine

Q5 How often do you experience migraine attacks?

- ◯ I have chronic migraine (15 days+ per month) (4)
- ◯ Once or twice per week (2)
- ◯ Once or twice per month (3)
- ◯ Once or twice per year (5)

Q6 When was the last time you experienced a migraine attack?

- ◯ This week (1)
- ◯ This month (2)
- ◯ During the last three months (3)
- ◯ This year (4)
- ◯ I don't remember (5)

Q7 How long do your migraine attacks typically last?

- ◯ Less than 30 min (2)
- ◯ 30 min to an hour (3)
- ◯ Over an hour (4)
- ◯ The whole day (5)
- ◯ Several days (6)

Q8 Have you ever been diagnosed with migraine by a medical professional?

- ◯ No (9)
- ◯ Yes, with Migraine (no aura) (2)
- ◯ Yes, with Migraine with aura (1)
- ◯ Yes, with Hemiplegic Migraine (3)
- ◯ Yes, with Abdominal Migraine (4)
- ◯ Yes, with Vestibular Migraine (6)
- ◯ Yes, with Cyclic Vomiting Syndrome (7)
- ◯ Yes, with Alice in Wonderland Syndrome (8)
- ◯ Yes, other type / I don't know the type (10) ____

© Imperial College of Science, Technology and Medicine

Q9 On a scale of 0-5, where 0 is not at all & 5 is extremely, having migraine disease affects:

	0 1 2 3 4 5
My physical wellbeing (e.g. experiencing pain or other symptoms) ()	
My emotional wellbeing (e.g. feeling anxious or desperate) ()	
My social wellbeing (e.g. having to cancel or avoiding social events) ()	

Q10 Select the activities you do for self-care maintenance for the **PREVENTION** of migraine attacks & to maintain physical & emotional wellbeing (Select all that apply)

- ▢ Keeping a headache diary (1)
- ▢ Keeping a mood diary (4)
- ▢ Lifestyle adaptations (e.g. regular breaks, avoiding screen time) (5)
- ▢ Counselling, Cognitive behavioural therapy, other mental health support (6)
- ▢ Taking over-the-counter medication (7)
- ▢ Taking prescription medication (8)
- ▢ Relaxation (e.g. meditation, breathing, progressive muscle relaxation, gentle yoga) (9)
- ▢ Exercise (yoga, stretching, massage, cardio) (10)
- ▢ Diet change and/or supplements (12)
- ▢ Staying hydrated (13)
- ▢ Migraine relief products (e.g. ice/heat pack, light blocking glasses) (14)
- ▢ Other (Please specify) (15) ____

© Imperial College of Science, Technology and Medicine

Q13 Please rate the extent that you think the following strategies are/ could be effective in PREVENTING a migraine attack.

	Probably ineffective (55)	No change (56)	Probably effective (57)
Keeping a headache diary (1)			
Keeping a mood diary (19)			
Lifestyle adaptations (e.g. regular breaks, avoiding screen time) (20)			
Counselling, Cognitive behavioural therapy, other mental health [illegible]			
Relaxation (e.g. meditation, breathing, progressive muscle relaxation, gentle [illegible]			
Exercise (Yoga, stretching, massage, cardio) (23)			
Diet change and/or supplements (25)			
Staying hydrated (26)			
Migraine relief products (e.g. ice/ heat pack, light blocking glasses) [illegible]			

© Imperial College of Science, Technology and Medicine

Q11 Select all the activities you do for self-care maintenance to **MANAGE** signs & symptoms of migraines when they occur (Select all that apply)

- ▢ Keeping a headache diary (1)
- ▢ Keeping a mood diary (4)
- ▢ Lifestyle adaptations (e.g. regular breaks, avoiding screen time) (5)
- ▢ Counselling, Cognitive behavioural therapy, other mental health support (6)
- ▢ Taking over-the-counter medication (7)
- ▢ Taking prescription medication (8)
- ▢ Relaxation (e.g. meditation, breathing, progressive muscle relaxation, gentle yoga) (9)
- ▢ Exercise (yoga, stretching, massage, cardio) (10)
- ▢ Diet change and/or supplements (12)
- ▢ Staying hydrated (13)
- ▢ Migraine relief products (e.g. ice/heat pack, light blocking glasses) (14)
- ▢ Other (Please specify) (15)

____

Q12 Please rate the extent that you think the following strategies are/ could be effective in **MANAGING** an acute migraine attack.

	Probably ineffective (8)	Unsure (9)	Probably effective (10)
Taking prescription medication (30)			
Taking over-the-counter medication (1)			
Relaxation (e.g. meditation, breathing, progressive muscle relaxation, gentle [illegible]			
Exercise (Yoga, stretching, massage, cardio) (32)			
Staying hydrated [illegible]			
Migraine relief products (e.g. ice/ heat pack, light blocking glasses) [illegible]			

© Imperial College of Science, Technology and Medicine

Q14
Do you feel that your mood or emotions (e.g. excitement or stress/anger) can be a trigger for your migraine attacks?

- Never (1)
- Sometimes (2)
- Most of the time (3)
- Always (4)

Display This Question:

If Do you feel that your mood or emotions (e.g. excitement or stress/anger) can be a trigger for you... = Most of the time

Or Do you feel that your mood or emotions (e.g. excitement or stress/anger) can be a trigger for you... = Always

Or Do you feel that your mood or emotions (e.g. excitement or stress/anger) can be a trigger for you... = Sometimes

Q15 What do you do to spot, manage or prevent these moods or emotional trigger(s)?

____

Q16 Do you keep track of your PHYSICAL wellbeing & symptoms?

- Yes (please explain how) (1) ____
- No (2)

Display This Question:

If Do you keep track of your PHYSICAL wellbeing & symptoms? = Yes (please explain how)

Q17 What do you do with the data you collect about your PHYSICAL wellbeing & symptoms?

- Identify triggers (1)
- Show reports to a healthcare professional (2)
- Understand when I have to take medication (3)
- Track improvements (4)
- Track deteriorations (5)
- Other (please specify) (6) ____

© Imperial College of Science, Technology and Medicine

Q18 Do you keep track of your EMOTIONAL wellbeing & symptoms?

◯ Yes (please explain how) (1) ____

◯ No (2)

Display This Question:

If Do you keep track of your EMOTIONAL wellbeing & symptoms? = Yes (please explain how)

Q19 What do you do with the data you collect about your EMOTIONAL wellbeing & symptoms?

▢ Identify triggers (1)

▢ Show them to a healthcare professional (2)

▢ Understand when I have to act to improve my emotional wellbeing (3)

▢ Track improvements (4)

▢ Track deteriorations (5)

▢ Other (please state) (6) ____

End of Block: Start page

Start of Block: Block 6 Demographics

Q20 What is your gender?

◯ Male (1)

◯ Female (2)

◯ Other (3)

*

Q21 How old are you (years)?

____

Q22 Where are you based?

◯ United Kingdom (4)

◯ United States (5)

◯ Germany (6)

◯ Austria (7)

© Imperial College of Science, Technology and Medicine

Display This Question:

If Where are you based? = United Kingdom

Q23 What is your ethnicity?

- ◯ White (1)
- ◯ Mixed/Multiple ethnic groups (25)
- ◯ Asian/ Asian British (26)
- ◯ British Black/African/Caribbean (27)
- ◯ Other ethnic group (please specify): (28) ____
- ◯ Would rather not say (29)

Display This Question:

If Where are you based? = United States

Q24 What is your ethnicity?

- ◯ White or European American (1)
- ◯ Black or African American (2)
- ◯ Asian American (4)
- ◯ American Indian/Alaska Native (5)
- ◯ Native Hawaiian/Other Pacific Islander (6)
- ◯ Multiracial American (3)
- ◯ Any other ethnic group, please describe (7) ____

Display This Question:

If Where are you based? = Germany

And Where are you based? = Austria

Q25 What is your ethnicity?

- ◯ European (1)
- ◯ Middle Eastern/Northern African (2)
- ◯ Sub-Saharan African (3)
- ◯ East Asian, Central Asian and South/Southeast Asian (4)
- ◯ Americas (5)
- ◯ Australia/Oceania (6)
- ◯ Any other ethnic group, please describe (7) ____

© Imperial College of Science, Technology and Medicine

Q26 What is your highest level of education?

- ◯ Secondary School (1)
- ◯ A levels/ College (2)
- ◯ University Degree or higher (3)

Q27 What is your employment status?

- ◯ Employed (1)
- ◯ Unemployed (2)
- ◯ Self-employed (3)
- ◯ Furloughed (4)
- ◯ Retired (5)
- ◯ Unable to work (7)

Q28 What is your marital status?

- ◯ Married (1)
- ◯ Single (8)
- ◯ In a domestic relationship (2)
- ◯ Divorced (4)
- ◯ Separated (5)
- ◯ Widowed (6)
- ◯ Other (please specify) (7) ____

Q29 Thank you for taking the time to answer our survey.
We are looking to interview up to 10-15 participants (via telephone, Skype or Microsoft Teams) to gain a deeper understanding of your experience with migraine and to create new services that may better help people manage their migraine and emotional wellbeing.

If you are interested in being contacted for an interview (approximately 30 minutes), please enter contact information and we will be in touch; if you are not interested please click next to register your responses. All information will be confidential.

- ◯ Name: (1) ____
- ◯ Email: (2) ____
- ◯ Mobile number: (3) ____

© Imperial College of Science, Technology and Medicine

End of Block: Block 6 Demographics

© Imperial College of Science, Technology and Medicine
